# A20 suppresses canonical Smad-dependent fibroblast activation: novel function for an endogenous inflammatory modulator

**DOI:** 10.1186/s13075-016-1118-7

**Published:** 2016-10-03

**Authors:** Swati Bhattacharyya, Wenxia Wang, Lauren Van Duyn Graham, John Varga

**Affiliations:** 1 Department of Medicine, Division of Rheumatology, Northwestern University Feinberg School of Medicine, McGaw M230, 240 E Huron Street, Chicago, IL 60611 USA; 2 Department of Dermatology, Northwestern University Feinberg School of Medicine, GALTER 676 N St Clair St Suite 1600 CH HNMH, Chicago, IL 60611 USA

**Keywords:** Systemic sclerosis, A20, TNFAIP3, TLR4, LPS, TGF-ß, Fibrosis, Type I collagen, IL-6, adiponectin

## Abstract

**Background:**

The ubiquitin-editing cytosolic enzyme A20, the major negative regulator of toll-like receptor (TLR)-mediated cellular inflammatory responses, has tight genetic linkage with systemic sclerosis (SSc). Because recent studies implicate endogenous ligand-driven TLR signaling in SSc pathogenesis, we sought to investigate the regulation, role and mechanism of action of A20 in skin fibroblasts.

**Method:**

A20 expression and the effects of forced A20 expression or siRNA-mediated A20 knockdown on fibrotic responses induced by transforming growth factor-ß (TGF-ß) were evaluated was evaluated in explanted human skin fibroblasts. Additionally, A20 regulation by TGF-ß, and by adiponectin, a pleiotropic adipokine with anti-fibrotic activity, was evaluated.

**Results:**

In normal fibroblasts, TGF-ß induced sustained downregulation of A20, and abrogated its TLR4-dependent induction. Forced expression of A20 aborted the stimulation of collagen gene expression and myofibroblast transformation induced by TGF-ß, and disrupted canonical Smad signaling and Smad-dependent transcriptional responses. Conversely, siRNA-mediated knockdown of A20 enhanced the amplitude of fibrotic responses elicited by TGF-ß. Adiponectin, previously shown to block TLR-dependent fibrotic responses, elicited rapid and sustained increase in A20 accumulation in fibroblasts.

**Conclusion:**

These results identify the ubiquitin-editing enzyme A20 as a novel endogenous mechanism for negative regulation of fibrotic response intensity. Systemic sclerosis-associated genetic variants of A20 that cause impaired A20 expression or function, combined with direct suppression of A20 by TGF-ß within the fibrotic milieu, might play a significant functional role in persistence of fibrotic responses, while pharmacological augmentation of A20 inhibitory pathway activity might represent a novel therapeutic strategy.

**Electronic supplementary material:**

The online version of this article (doi:10.1186/s13075-016-1118-7) contains supplementary material, which is available to authorized users.

## Background

Systemic sclerosis (SSc) is characterized by autoimmunity, vascular injury and tissue fibrosis [1]. Fibroblast activation resulting in collagen overproduction and myofibroblast differentiation plays a central role in the development and progression of fibrosis in the skin and internal organs. The multifunctional cytokine transforming growth factor-ß (TGF-ß) is a potent stimulus for fibroblast activation, and is strongly linked to the pathogenesis of SSc [2]. Although multiple intracellular pathways have been implicated in TGF-β-mediated fibrotic responses, cross-regulation among these networks remain incompletely characterized. Moreover, the factors underlying persistence of pathological inflammation and fibrosis in SSc are largely unknown. Recent experimental studies and results from genetic and genomic surveys provide compelling evidence to link innate immune signaling and toll-like receptor (TLR) activation to TGF-β activity and persistent fibrotic response [3].

Toll-like receptors are evolutionary conserved cellular sensors recognizing both microbial (exogenous) pathogen-associated molecular patterns (MAMPs) and endogenous “damage-associated molecular patterns” (DAMPs) [3, 4]. Deregulated or unchecked TLR activation leads to persistent and unresolving tissue damage. Recent studies provide evidence for an essential pathogenic role for TLR4 and endogenous TLR ligands in SSc [5]. In order to avoid injury resulting from aberrant or sustained innate immune responses, a number of cellular mechanisms have evolved that negatively regulate TLR signaling [3, 6]. Best studied of these to date is the ubiquitin-editing enzyme A20 (TNFAIP3), a direct downstream target of TLR signaling, which is rapidly and transiently induced by lipopolysaccharide (LPS), and negatively regulates both TLR-dependent and TLR-independent inflammatory responses [7].

Genetic association studies have uncovered tight linkage of single nucleotide polymorphisms (SNPs) in the A20 locus with susceptibility to autoimmune and inflammatory diseases, including SSc, in multiple ethnic cohorts [8–10]. Whereas the key role of A20 in modulating TLR4 activity and target gene expression in immune cells such as macrophages and dendritic cells is now quite well-established, the regulation, mode of action and importance of A20 in stromal cells such as fibroblasts remain completely unknown. Furthermore, the expression and function of A20 in the context of physiologic and pathological matrix remodeling have not been explored to date. The genetic linkage of A20 variants with SSc susceptibility and the growing recognition of a pathogenic role for TLR signaling in fibrosis and SSc, coupled with the scleroderma-like skin phenotype described in A20 knockout mice [11] together suggest a likely important role for A20 in the pathogenesis of SSc.

In the present studies we demonstrate show that A20 is detectable in normal skin fibroblasts, and shows sustained downregulation in response to TGF-β. Forced transient A20 expression in fibroblasts resulted in suppression of fibrotic responses elicited by TGF-ß and TLR4 ligands. These inhibitory effects of A20 were accompanied by reduced amplitude of canonical TGF-ß signaling and Smad-dependent transcriptional responses. These results reveal an important novel physiologic role for A20 in negatively regulating the intensity of fibrotic responses in fibroblasts, which parallels its role in limiting inflammatory responses. Impaired tissue expression or function of A20 due to SSc-associated genetic variants, combined with environmental regulatory influences, might contribute directly to the development or progression of fibrosis in SSc.

## Methods

### Cell culture and reagents

Primary cultures of dermal fibroblasts were established by explantation from neonatal foreskin or adult skin biopsies as described [12]. Cultures were maintained in Dulbecco’s Modified Eagle’s medium (DMEM) supplemented with 10 % fetal calf serum (FCS) (Gibco BRL, Grand Island, NY, USA), 1 % vitamin solutions and 2 mM L-glutamine. All other tissue culture reagents were from Lonza (Basel, Switzerland). For experiments, low-passage (3–5) fibroblasts were placed in serum-free media containing 0.1 % bovine serum albumin (BSA) for 24 h prior to addition of TGF-ß2 (PeproTech, Rocky Hill, NJ, USA), which has overlapping activity with TGF-ß1 and has been widely used to study the modulation of fibroblast activity (12,14). In other experiments, fibroblasts were incubated with recombinant human full-length adiponectin (BioVendor, Karasek, Czech Republic) or ultrapure LPS (InvivoGen, San Diego, CA, USA).

### Isolation and analysis of RNA

Total RNA, isolated from fibroblasts cultures, was reverse-transcribed to complementary DNA (Cdna) using qScript cDNA SuperMix (Quanta Bio-Sciences, Gaithersburg, MD) and analyzed by real-time qPCR [12, 13]. Products (20 ng) were amplified using SYBR Green PCR Master Mix (Applied Biosystems, Grand Island, NY, USA) using an Applied Biosystems 7500 Prism Sequence Detection System. Results were normalized to glyceraldehyde-3-phosphate dehydrogenase (GAPDH) RNA levels, and fold change was calculated in the samples [12].

### Western analysis

At the end of each experiment, fibroblasts were harvested and whole-cell lysates subjected to western analysis as described [12]. The following antibodies were used: anti-phospho-Smad2 (Cell Signaling Technology, Boston, MA, USA), anti-type I collagen (Southern Biotech, Birmingham, AL, USA), A20 (Santa Cruz, Dallas, TX, USA), anti-α smooth muscle-actin (αSMA), beta actin and tubulin (all from Sigma, St. Louis, MO, USA). Proteins were visualized using ECL reagents (Pierce, Rockford, IL, USA) and levels were quantitated by determining band intensities normalized to loading controls in each lane using ImageJ software.

### Immunofluorescence confocal cytochemical analysis

Fibroblasts seeded on 8-well Lab-Tek II chamber glass slides (Nalgene Nunc International, Naperville, IL, USA) were incubated with TGF-ß (10 ng/ml) or transfected with A20 (Addgene, Cambridge, MA, USA) in serum-free DMEM with or without TGF-ß2 (Peprotech, Rocky Hill, NJ, USA) (10 ng/ml) for up to 24 h. Cells were then fixed, permeabilized, and incubated with anti-αSMA (1:100) (Sigma), anti-Smad2/3 (Cell Signaling Technology) and anti-A20 (1:100) (Santa Cruz) antibodies, followed by Alexa-fluor-labeled secondary antibodies (Invitrogen). Nuclei were identified using 4,6-diamidino-2-phenylindone (DAPI) and immunofluorescence was evaluated under the Nikon A1 confocal microscope.

### Transient transfection assays

Fibroblasts at early confluence were transfected with A20 or empty vector using Lipofectamine®2000 Reagent (ThermoFisher Scientific, Grand Island, NY, USA) in parallel [14]. Cultures were incubated in serum-free media containing 0.1 % BSA for 24 h, followed by TGF-ß2 for a further 24 h. At the end of the experiments, cultures were harvested and whole-cell lysates were prepared, or fibroblasts were fixed and subjected to immunofluorescence confocal microscopy. In selected experiments, fibroblasts were transiently transfected with [SBE]_4_-luc plasmid along with Renilla luciferase pRL-TK (Promega) as control for transfection efficiency. Following a 24-h incubation, whole-cell lysates were prepared and assayed for their luciferase activity.

### Small interfering RNA-mediated knockdown

Fibroblasts were transfected with target-specific small interfering RNAs (siRNAs) (10 nM) coding for A20 or scrambled control siRNA (Dharmacon, Lafayette, CO, USA). At 24 h following transfection, fresh media containing TGF-ß2 (10 ng/ml) were added to the cultures, and incubations continued for a further 24 h, when total RNA was isolated for analysis.

### Statistical analysis

Data are presented as means ± SD unless otherwise indicated. Differences between two groups were determined by Student’s *t* test. A *p* value <0.05 was considered statistically significant. Comparisons among three or more groups were performed using analysis of variance (ANOVA) followed by Sidak’s correction for multiple comparisons. Data were analyzed using Graph Pad prism (Graph Pad Software version 6, Graph Pad Software Inc., CA, USA).

## Results

### A20 is detected in skin fibroblasts and its basal and inducible expression is suppressed by TGF-ß

**Fig. 1:**
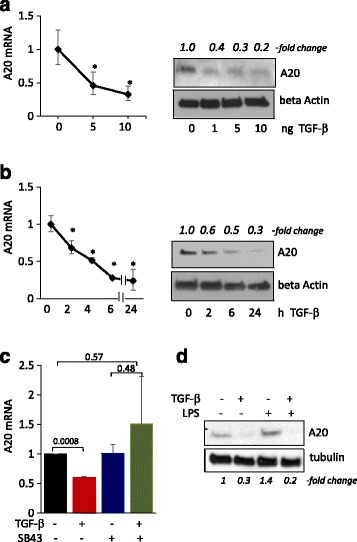
Transforming growth factor-ß (*TGF-ß*) down-regulated basal A20 expression and prevented its induction. Confluent foreskin fibroblasts were incubated with TGF-ß2 (10 ng/ml or indicated concentrations) or ultrapure lipolysaccharide (*LPS*) (500 ng/ml) for 24 h or indicated periods. RNA and whole cell lysates were examined by qPCR (**a**, **b** (*left panels*), **c**) and western analysis (**a**, **b** (*right panels*), **d**). Representative immunoblots (*n* = 3). Band intensities, normalized for beta actin or tubulin in each lane are shown below. qPCR results, normalized with glyceraldehyde-3-phosphate dehydrogenase (GAPDH), are means ± SD of triplicate determinations (*n* = 3); *p < 0.05

While constitutive A20 expression is low in most normal cell types, A20 was detectable in cultured human fibroblasts in the absence of stimulation [7]. In light of the central role of TGF-ß in modulating pathogenic fibroblast responses in SSc, we sought to examine the possibility that it might modulate the expression of A20. To this end, confluent foreskin fibroblasts were incubated with TGF-ß. The results demonstrated that TGF-ß treatment induced a dose-dependent and time-dependent decrease in A20 gene expression, with maximal inhibition after 24 h (Fig. 1a and b). Comparable results were seen when normal adult skin fibroblasts were used (Additional file 1: Figure S1 and data not shown). To investigate the cellular mechanisms underlying suppression of A20 by TGF-ß, we examined the effect of SB43542, a potent and selective inhibitor of ALK5 receptor-mediated Smad2/3 phosphorylation. The results showed that pretreatment with ALK5 inhibitor substantially reduced the suppressive effect of TGF-ß on A20 expression (Fig. 1c), indicating a key role for canonical Smad signaling in mediating this inhibitory effect of TGF-ß on A20.

Expression of A20 is rapidly and transiently induced by the prototypic TLR4 ligand LPS, and one of the best characterized roles of A20 is negative regulation of TLR signaling in an inhibitory feedback loop [6]. We had shown previously that in normal fibroblasts LPS by itself, or together with TGF-ß, elicits TLR4-dependent fibrotic responses [14]. To explore whether LPS induction of A20 is modulated by TGF-ß, fibroblasts were incubated with LPS [15], in the presence or absence of TGF-ß. While LPS enhanced A20 expression as expected, stimulation was completely abolished by pretreatment of the cultures with TGF-ß, indicating a dominant inhibitory role for TGF-ß in the regulation of basal and inducible A20 expression (Fig. 1d).

### A20 abrogates TGF-ß-induced fibrotic responses in skin fibroblasts

**Fig. 2:**
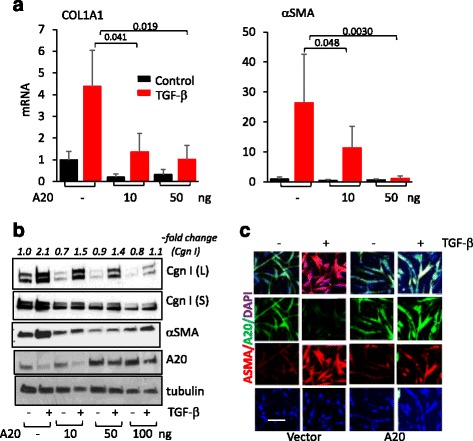
A20 attenuates fibrotic responses elicited by transforming growth factor-ß (*TGF-ß*). Foreskin fibroblasts transiently transfected with A20 or empty vector were incubated with TGF-ß2 (10 ng/ml) for 24 h and harvested. RNA, whole cell lysates and media were isolated, and examined by qPCR (**a**) and western analysis (**b**). qPCR results, normalized with glyceraldehyde-3-phosphate dehydrogenase (GAPDH), are means ± SD of triplicate determinations (*n* = 3). *Cgn I* Type I collagen, *L* cell lysates, *S* secreted. Representative immunoblots (*n* = 3). Band intensities, normalized for tubulin in each lane are shown below. **c** Immunofluorescence confocal microscopy using antibody to αSMA (*red*). Nuclei identified by 6-diamidino-2-phenylindone (*DAPI*) (*blue*). *Bar* = 25 μm

**Fig. 3:**
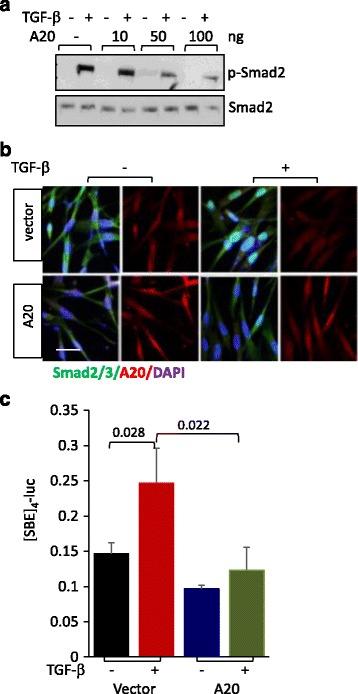
A20 blocks intracellular canonical transforming growth factor-ß (*TGF-ß*)-Smad signaling. Foreskin fibroblasts transiently transfected with A20 or empty vector were incubated with TGF-ß2 (10 ng/ml) for 24 h. **a** Whole cell lysates were examined by western analysis (*n* = 2). **b** Immunofluorescence confocal microscopy using antibody to Smad2/3 (green color). Nuclei identified by 6-diamidino-2-phenylindone (*DAPI*) (*blue*). *Bar* = 25 μm. **c** Fibroblasts were transiently co-transfected with [SBE]_4_-luc and A20 (50 ng) in absence or presence of TGF-ß2 along with Renilla luciferase pRL-TK. Following a 24-h incubation, whole-cell lysates were collected and assayed for their luciferase activity. Results are means ± SD of triplicate determinations (*n* = 2)

While A20 has been convincingly implicated in negative regulation of nuclear factor (NF)-kB-mediated inflammatory responses in a variety of cell types [6, 16], its potential impact on modulating fibrotic responses in fibroblasts has not been explored. We therefore investigated the effect of A20 in transiently transfected human fibroblasts. While TGF-ß caused marked suppression of A20, confirming our earlier observations, forced expression of A20 in these cells attenuated TGF-ß-induced stimulation of collagen and α-smooth muscle actin (αSMA) gene expression and myofibroblast differentiation in a dose-dependent manner (Fig. 2a-c). To investigate the mechanisms underlying these anti-fibrotic effects, we focused on canonical Smad signaling, which is recognized as a fundamental pathway driving fibrotic TGF-ß responses [2]. TGF-ß-induced rapid stimulation of Smad2 phosphorylation and nuclear localization were substantially attenuated by ectopic A20 expression in these fibroblasts (Fig. 3a and b). Moreover, transient transfection assays indicated attenuated stimulation of [SBE]_4_-luc activity in fibroblasts with A20 overexpression (Fig. 3c). Together, these findings demonstrate a novel function for A20 as a potent endogenous inhibitor of Smad-dependent fibrotic responses in fibroblasts.

### Ectopic A20 attenuates LPS-induced fibrotic responses in skin fibroblasts

**Fig. 4:**
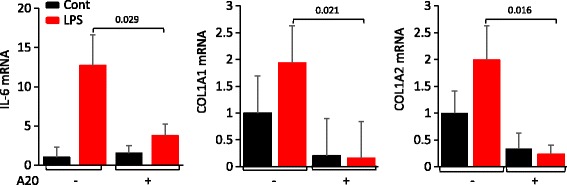
A20 attenuates toll-like receptor 4 (TLR4)-dependent fibrotic responses. Fibroblasts transiently transfected with A20 or empty vector were incubated with ultrapure lipolysaccharide (*LPS*) and/or transforming growth factor-ß2 (TGF-ß2) (10 ng/ml). Following a 24-h incubation, RNA isolated and was examined by qPCR; results, normalized with glyceraldehyde-3-phosphate dehydrogenase (GAPDH), are means ± SD of triplicate determinations (*n* = 2)

In light of the previously established profibrotic effects of TLR4 [14], in complementary experiments we sought to examine the modulation of these responses by A20. For this purpose, confluent fibroblasts expressing ectopic A20 were incubated with LPS and/or TGF-ß. Forced expression of A20 completely abrogated LPS-induced stimulation of type I collagen synthesis, COL1A1 and COL1A2 mRNA expression, and myofibroblast differentiation. These results indicate that A20 blocked TLR4-dependent fibrotic responses in a manner similar to its inhibitory effects on TLR4-dependent inflammation (Fig. 4). Indeed, LPS-induced stimulation of interleukin-6 (IL-6) was completely abrogated by A20.

### Suppression of fibroblast A20 is associated with enhanced fibrotic responses

**Fig. 5:**
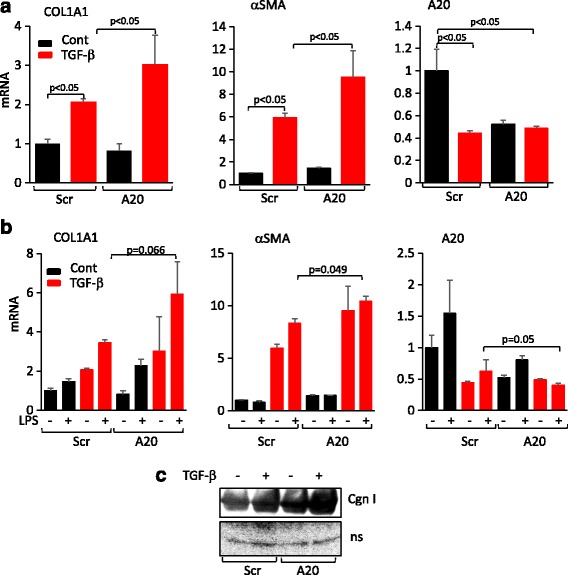
Fibrotic responses are negatively regulated by cellular A20. Foreskin fibroblasts were transfected with A20-specific siRNA or scrambled (*Scr*) control, followed by incubation with transforming growth factor-ß2 (*TGF-ß2*) (10 ng/ml), lipopolysaccharide (LPS) (500 ng/ml) or both for 24 h. **a**, **b** mRNA levels were determined by real-time qPCR. Results, normalized with glyceraldehyde-3-phosphate dehydrogenase (GAPDH), are means ± SD of triplicate determinations (*n* = 2). **c** Western analysis of secreted Type I collagen (*Cgn I*). *ns* nonspecific band

These results suggested that A20 might have a novel cell-autonomous negative regulatory role in fibrotic responses. To directly evaluate this possibility, we used an RNAi approach. Transfection of foreskin fibroblasts with A20 siRNA caused a substantial reduction in cellular A20 levels that was accompanied by significantly enhanced stimulation of collagen and αSMA gene expression elicited by TGF-ß (Fig. 5a and c). Moreover, A20 knockdown enhanced both LPS and TGF-ß-induced stimulation of type I collagen synthesis, COL1A1 and COL1A2 mRNA expression, and myofibroblast differentiation (Fig. 5b). These results point to a hitherto-unrecognized cell-autonomous function of A20 in repressive modulation of fibrotic responses elicited by TGF-ß or TLR4 ligands.

### Adiponectin stimulates the expression of A20

**Fig. 6:**
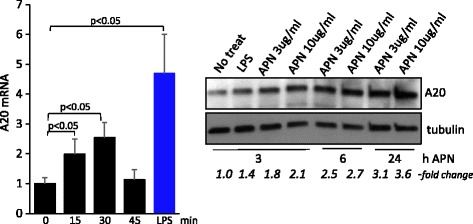
Adiponectin induces A20 gene expression in fibroblasts. Foreskin fibroblasts were incubated with ultrapure lipolysaccharide (*LPS*) (500 ng/ml, 30 minutes) (*lane 5*, *left panel*) and/or recombinant adiponectin (*APN*) (10 μg/ml in *left panels* or as indicated in the *right panel*) for 0–45 minutes or as indicated. *Left panel*, levels of RNA determined by qPCR. Results, normalized with glyceraldehyde-3-phosphate dehydrogenase (GAPDH), are means ± SD of triplicate determinations (*n* = 2). *Right panels*, western analysis of whole-cell lysates. Representative immunoblots (*n* = 2). Band intensities, normalized for tubulin in each lane are shown below

Adipokines secreted from white adipose tissue exert pleiotropic local and hormonal effects and play key roles in regulating metabolism, inflammation and tissue repair (13). Adiponectin is unique among adipokines in that it is decreased in obese humans and experimental animals [17, 18]. Adiponectin is secreted from, and targets, not only adipocytes, but also macrophages and resident stromal cells. Transcriptional profiling in macrophages previously revealed that one of the top genes upregulated by adiponectin was A20 [15]. We had shown earlier that treatment of fibroblasts with adiponectin was associated with potent anti-fibrotic effects [19]. To investigate whether adiponectin might have an effect on modulating the expression of A20 in skin fibroblasts, confluent monolayers were incubated with recombinant full-length human adiponectin for various periods. The results showed that adiponectin treatment in these cells resulted in rapid upregulation of A20 mRNA, with increased levels as early as 15 minutes (Fig. 6). Increased A20 protein levels were detected in these cells at 3 h and were persistently elevated for up to 24 h.

## Discussion

Persistent fibrosis in multiple organs, the hallmark of SSc, is due to excessive extracellular matrix (ECM) secretion and matrix stiffening driven by metabolically activated myofibroblasts. While growth factors and morphogens such as TGF-ß, platelet-derived growth factor (PDGF) and Wnt ligands have been broadly implicated in triggering fibroblast activation, the mechanism responsible for maintaining persistent activation in pathological tissue repair remain poorly understood [5]. The ubiquitin-editing enzyme A20 inhibits NF-kB-dependent responses, and alterations in its expression or function are implicated in a variety of chronic inflammatory and malignant conditions [7]. In light of the multi-ethnic genetic association of several SNPs of A20/TNFAIP3 with SSc [10, 20], the present studies sought to investigate the potential regulatory function of A20 in modulating fibroblast responses.

Our results show, for the first time, that while A20 is detectable in unstimulated skin fibroblasts, treatment with TGF-ß caused marked suppression and prevented its induction by LPS. Moreover, A20 exerted a potent cell-autonomous inhibitory effect on fibrotic gene expression, and reducing cellular A20 levels was associated with accentuated fibrotic responses and Smad2/3 activation. The results indicate that A20 is capable of antagonizing intracellular TGF-ß/Smad signaling. In light of the frequent genetic alterations in the A20 locus in SSc patients leading to altered A20 expression and/or regulatory function, our findings suggest that unchecked TGF-ß signaling and persistent fibrosis in SSc might result from impaired A20 in SSc fibroblasts. Conversely, pharmacologic rescue of A20 in SSc might restore appropriate regulation of fibrotic processes, and could therefore represent a novel approach to treatment.

A20 is a dual function ubiquitin-editing enzyme with both deubiquitinase and E3 ubiquitin ligase activities [7]. A20 is most prominently implicated in regulating the duration and intensity of inflammation, but an expanding range of biological processes is recognized as modulated by A20. By inhibiting tumor necrosis factor receptor-associated factor (TRAF6) ubiquitin ligase, a critical node in proinflammatory signal transduction, A20 prevents recruitment of TAK1 and activation of NF-kB [16]. Additionally, A20 also has been shown to inhibit inflammatory signaling downstream of the IL-1 receptor, TNF receptor, Nod-like receptor, CD40 and most importantly, TLRs [21]. The expression of A20 is itself positively regulated by these inflammatory signals via NF-kB; thus, induction of A20 constitutes a critical negative feedback loop for NF-kB signaling.

Recent studies indicate that the deubiquitinase activity of A20 is not absolutely required for controlling NF-kB signaling [22]. A20-deficient cells show sustained NF-kB activation and enhanced inflammatory cytokine production in response to TNF and TLR ligands. Mouse models provide compelling evidence for the critical role of A20 in modulating inflammatory responses. Global deletion of A20 is associated with premature mortality due to spontaneous multi-organ inflammation [11], while partial loss in haplo-insufficient mice is associated with impaired tissue regeneration and spontaneous neuroinflammation [23, 24]. Conditional ablation of A20 in myeloid cells results in spontaneous arthritis, while ablation of A20 in dendritic cells causes autoimmune disease with lupus-like and inflammatory bowel disease features [6]. Mice with A20 ablation in keratinocytes manifest psoriasis-like epidermal hyperproliferation and disheveled hair in the absence of inflammation [25]. Several lymphoma types are associated with somatic mutations and chromosomal deletions in the TNFAIP3 locus, and show reduced A20 expression or activity [26]. Genetic studies have linked germline SNP variants to susceptibility to a variety of autoimmune and auto-inflammatory diseases along with SSc [6]. Many of these genetic variants cause reduced expression or activity of A20.

Recent studies implicate A20 in the pathogenesis of SSc. Strong association of SSc with a SNP within intron 2 (rs5029939) of A20 was found, particularly in patients with diffuse cutaneous involvement and fibrosing alveolitis [27]. In a subsequent genome-wide association study (GWAS) SSc was also associated with the non-synonymous coding variant rs2230926 [28]. This SNP, the most common A20 coding SNP identified to date in autoimmune diseases, causes a phenylalanine-to-cysteine change at residue 127, resulting in a protein with impaired NF-kB inhibitory activity [29].

In addition to the genetic evidence, further interest in the A20-SSc link comes from recent studies indicating a pathogenic role of aberrant TLR signaling in SSc [3]. The expression of both TLR4 and of its DAMP endogenous ligands is markedly enhanced in SSc skin and lung tissue [12, 30]. Moreover, TLR4 activation on stromal cells elicits sustained fibrotic signaling, and contributes to failure to resolve experimental fibrosis in mice [14]. The TLR4-dependent fibrotic response is further amplified in the presence of TGF-ß [14], suggesting a pathogenic role for the TLR4 ligand-receptor signaling axis in SSc. Computational gene network analysis showed that A20 serves as a central hub for inflammatory-fibrotic networks in skin lesions in SSc [31]. In view of the consistent genetic associations of A20 SNPs with disease susceptibility, its fundamental role in constraining TLR4 signaling, and the central location of A20 in tissue-specific gene networks in SSc, it was of substantial interest to examine expression, regulation and function of A20 in the context of fibrogenesis.

Expressed in most cell types at low or undetectable levels, A20 is induced rapidly and transiently by inflammatory signals [7]. In autoimmune diseases associated with A20 SNPs there is reduced NF-kB binding to the A20 promoter, attenuating A20 expression [32]. Epigenetic modifications such as histone acetylation or promoter methylation, might further contribute to reduced A20 expression or induction, as has been shown in peripheral blood monocytes in systemic lupus erythematosus (SLE) [33] and B cell lymphoma [34]. We found that treatment of normal fibroblasts with TGF-ß resulted in time-dependent and dose-dependent repression of A20. Moreover, in the presence of TGF-ß, LPS treatment of fibroblasts failed to elicit maximal induction of A20. While the precise mechanisms underlying negative regulation of A20 by TGF-ß are currently unknown, these observations provide evidence that excessive local TGF-ß activity within the fibrotic milieu, a hallmark of SSc [35], might directly suppress A20. Future studies will be needed to precisely delineate the reciprocally antagonistic relationship between TGF-ß activity and A20 expression in SSc.

We found that A20 exerts potent negative effects on the expression of collagen, αSMA and related genes involved in fibrosis. The inhibitory response involved disruption of receptor-dependent Smad2/3 activation, the canonical signaling pathway primarily responsible for pathological fibroblast activation in fibrosis [5]. In fibroblasts overexpressing A20, stimulation with TGF-ß resulted in attenuated Smad2/3 phosphorylation, nuclear localization, and induction of Smad-dependent transcriptional responses. Therefore, there appears to be a reciprocal negative cross-regulatory relationship between TGF-ß and A20, with TGF-ß suppressing basal and inducible A20 expression, and A20 blocking Smad-dependent fibrotic responses.

We had shown previously that LPS exerts TLR4-dependent profibrotic effects mediated, at least in part, through suppressing miR29, which is an endogenous negative regulator of fibrotic responses (14). It is noteworthy that miR29 itself augments A20 expression, due to its competition with the RNA-degrading HuR and prevention of HuR-mediated transcript decay [36]. Whether A20 modulates the regulation of miR29, and if such regulation might be potentially implicated in the anti-fibrotic effects of A20, remain to be investigated. RNAi experiments indicated that A20 plays a cell-autonomous negative regulatory role in fibrotic gene expression, and in preventing excessive or prolonged stimulation of these genes in response to TGF-ß. It is of interest, in this regard, that mice with global A20 deletion were found to have a scleroderma-like phenotype with marked thickening of the dermis and disappearance of the intradermal white adipose tissue [11]. These findings, consistent with unchecked activation of dermal fibroblasts, are pathological hallmarks of lesional skin changes in SSc [1]. In view of these observations, we propose that enhancing A20 expression or activity in fibroblasts might be a potential approach to limiting excessive fibrotic signaling elicited by TGF-ß, and perhaps by other fibrotic stimuli such as Wnt/ß-catenin [37]. In this regard, it is of great interest that adiponectin, which has both anti-inflammatory and anti-fibrotic activity [18], elicited sustained A20 induction in fibroblasts. Augmentation of A20 is likely to account for the mitigation by adiponectin of TLR4-mediated inflammatory and oxidative responses. While it is currently unknown how adiponectin induces A20, and whether endogenous A20 contributes to the anti-fibrotic effects elicited by adiponectin, therapeutic targeting of the adiponectin-A20 axis using peptide agonists of the AdipoR1/R2 adiponectin receptors, might be feasible [38].

Taken together, the present findings identify a potentially important novel regulatory role for A20, a pervasive genetic risk for SSc susceptibility, in connective tissue homeostasis and fibrosis. Our results also establish a potential mechanism for controlling A20 expression by TGF-ß, which represses A20 in fibroblasts and prevents A20 induction via TLR4. Moreover, the results indicate that A20 mitigates fibrotic responses induced by TGF-ß, and through TLR4 activation, while A20 appears to be important as a cell-autonomous mechanism for restraining the amplitude or duration of fibrotic signaling. These findings implicate A20 as a critical endogenous rheostat for fibroblast activation and of cutaneous fibrogenesis.

Appropriate induction of A20 in the skin is likely to be important in maintaining tight spatial and temporal regulation of fibrotic gene responses during wound healing, and a protective role in limiting excessive scar formation. These findings are broadly in line with a growing recognition of A20 as a pleiotropic guardian of tissue homeostasis by virtue of its combined anti-inflammatory, anti-apoptotic, anti-oxidant and anti-fibrotic/pro-regenerative activities [39]. Augmenting A20 expression or function of A20 within injured tissue microenvironments might limit ongoing fibrogenic signaling, attenuate fibrosis and promote regeneration. Accordingly, pharmacologic agents that induce A20 expression such as vitamin E (γ-tocotrienol) and MALT inhibitors, along with adiponectin, hold promise as anti-fibrotic therapies via restoring endogenous A20 expression, particularly when targeted in a tissue-restricted manner, such as topical application to the skin.

## Conclusions

The present findings identify an important novel physiologic role for the cytosolic deubiquitinase A20 in fibrosis. Our results also establish a mechanism for controlling A20 expression by TGF-ß, which represses A20 in fibroblasts and prevents induction via TLR4. Furthermore, the results indicate that A20 alleviates fibrotic responses induced by TGF-ß, and through TLR4. These finding altogether implicate A20 as a critical cell-intrinsic modulator of fibroblast activation and of cutaneous fibrogenesis. Induction of A20 is likely to serve an important function in physiologic regulation of cutaneous fibrosis gene responses during wound healing. Impaired A20 expression or function due to genetic variants, epigenetic modulation and/or environmental influences might contribute directly to the development or progression of fibrosis in SSc, and represents a promising target for therapy.

## Additional file

Additional file 1: Figure S1. TGF-ß suppresses A20 expression in adult skin fibroblasts. Confluent adult skin fibroblasts were incubated with TGF-ß2 (10 ng/ml) for indicated time-points. **A** RNA was examined by qPCR. Results, normalized with GAPDH, are means ± SD of triplicate determinations (*n* = 2). **B** Western analysis of whole-cell lysates. Representative immunoblots (*n* = 2). Band intensities, normalized for beta actin in each lane are shown below (PDF 50 kb)
