# Supplementary figures and images for: A20 suppresses canonical Smad-dependent fibroblast activation: novel function for an endogenous inflammatory modulator
Source: Arthritis Res Ther. 2016 Oct 3;18:216. doi: 10.1186/s13075-016-1118-7 (PMC5048449; doi:10.1186/s13075-016-1118-7)

## Supplementary Figure 1

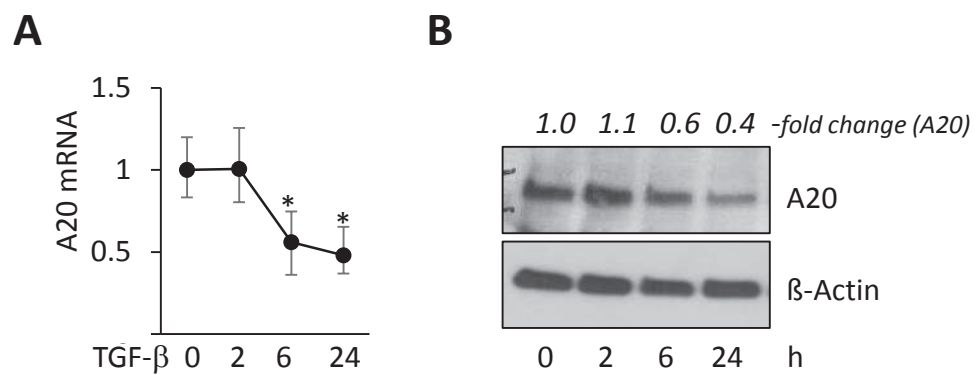

Supplement: Additional file 1: Figure S1. — TGF-ß suppresses A20 expression in adult skin fibroblasts. Confluent adult skin fibroblasts were incubated with TGF-ß2 (10 ng/ml) for indicated time-points. A RNA was examined by qPCR. Results, normalized with GAPDH, are means ± SD of triplicate determinations (n = 2). B Western analysis of whole-cell lysates. Representative immunoblots (n = 2). Band intensities, normalized for beta actin in each lane are shown below (PDF 50 kb) [file 13075_2016_1118_MOESM1_ESM.pdf]
